# Mode of presentation and performance of serology assays for diagnosing celiac disease: A single-center study in the United Arab Emirates

**DOI:** 10.3389/fnut.2023.1107017

**Published:** 2023-04-05

**Authors:** Abdullah Shatnawei, Asma H. AlNababteh, Romona Devi Govender, Saif Al-Shamsi, Ammar AlJarrah, Rami H. Al-Rifai

**Affiliations:** ^1^Cleveland Clinic Abu Dhabi, Abu Dhabi, United Arab Emirates; ^2^College of Medicine and Health Sciences, Institute of Public Health, United Arab Emirates University, Al Ain, United Arab Emirates; ^3^Department of Family Medicine, College of Medicine and Health Sciences, United Arab Emirates University, Al Ain, United Arab Emirates; ^4^Department of Internal Medicine, College of Medicine and Health Sciences, United Arab Emirates University, Al Ain, United Arab Emirates; ^5^Clinical Medical Science, Faculty of Medicine, Yarmouk University, Irbid, Jordan; ^6^Zayed Center for Health Sciences, United Arab Emirates University, Al Ain, United Arab Emirates

**Keywords:** celiac disease, serologic biomarkers, gastroenterology, duodenal biopsy, Cleveland Clinic, United Arab Emirates

## Abstract

**Objective:**

To characterize patients with celiac disease (CD), examines the clinical spectrum of CD, and evaluate the performance of serologic tests used for CD screening, in the United Arab Emirates (UAE).

**Methods:**

Medical charts of patients received at the Digestive Diseases Institute of Cleveland Clinic Abu Dhabi from January 2015 to December 2020 were reviewed. Patients who were screened for four serologic biomarkers (anti-tissue transglutaminase IgA [Anti-tTG-IgA], anti-tissue transglutaminase IgG [Anti-TtG-IgG], anti-deamidated gliadin peptide IgG [Anti-DGP-IgG], and anti-deamidated gliadin peptide IgA [Anti-DGP-IgA]) were included. Histopathology was performed on patients with the seropositive test. Marsh score > 1 considered to confirm CD. Characteristics of the Anti-tTG-IgA seropositive patients were described and that correlated with histopathologically confirmed CD were explored.

**Results:**

Of the 6,239 patients, 1.4, 2.9, 4.7, and 4.9%, were seropositive to Anti-tTG-IgG, Anti-TtG-IgA, Anti-DGP-IgA, and Anti-DGP-IgG, respectively. Overall, 7.7% were seropositive to either of the four biomarkers. Of the biopsy-screened 300 patients, 38.7% (1.9% of the total serologically screened) were confirmed with CD. The mean age of Anti-TtG-IgA seropositive patients was 32.1 ± 10.3 SD years, 72% of them were females, and 93.4% were Emirati. In those patients, overweight (28.7%) and obesity (24.7%) were common while 5.8% of patients were underweight. Anemia prevalence was 46.7%, 21.3% had Gastroesophageal reflux disease (GERD), 7.7% with autoimmune thyroid disease, 5.5% (type 1), and 3.3% (type 2) were diabetic. Vitamin D deficiency was observed in 47.8% of the Anti-TtG IgA seropositive patients. Twelve (10.3%) histopathologically confirmed CD patients were seronegative to Anti-TtG-IgA but seropositive to anti-DGP-IgA and/or Anti-DGP-IgG. Body mass index, GERD, autoimmune thyroid disease, type 1 diabetes, asthma, hemoglobin, and vitamin D concentration, were all correlated with biopsy-confirmed CD (*P* < 0.05). Compared to the gold-standard biopsy test, Anti-TtG-IgA had the highest sensitivity (89.7%) and specificity (83.7%).

**Conclusion:**

Three and two of every 100 patients were serologically (anti-tTG-IgA positive) and histopathologically diagnosed with CD, respectively. Although Anti-TtG-IgA is the most sensitive, specific, and commonly used test, one of every ten histopathologically confirmed patients and Anti-tTG-IgA seronegative were seropositive to Anti-DGP. To avoid missing patients with CD, a comprehensive serological investigation covering DGP-IgG/IgA is warranted.

## Introduction

Celiac disease (CD) is a chronic autoimmune gastrointestinal disorder in which dietary gluten enhances the immune response in genetically susceptible patients (i.e., those with human leukocyte antigen (HLA)-DQ2 or -DQ8 haplotypes) (1). In patients with CD, the ingestion of gluten causes varying degrees of inflammatory damage to the mucosa of the small intestine, leading to nutrient malabsorption (2). According to results of a systematic review by Singh et al. (3), the global prevalence of CD based on serologic markers and biopsy results is estimated at 1.4 and 0.7%, respectively. The biopsy-proven prevalence of CD is high in Europe and Oceania (0.8%) and low in South America (0.4%) and is higher in females (0.6%) than in males (0.4%) and in children (0.9%) as compared to adults (0.5%) (3). According to previous research, CD is closely associated with other autoimmune endocrine diseases including type 1 diabetes and autoimmune thyroid disease (4, 5). Thus, several international guidelines recommend the screening of CD in patients with type 1 diabetes and/or autoimmune thyroiditis (6, 7).

Patients susceptible to CD usually present with various symptoms due to malabsorption following histopathologic changes and damage to the duodenal mucosa. Possible clinical and subclinical symptoms include weight loss, diarrhea, steatorrhea, abdominal distension, iron deficiency anemia, osteoporosis, neurologic disease, non-specific abdominal symptoms, dermatitis herpetiformis, or malignancies (2), with the severity of symptoms varying between adults and children (8).

Although duodenal biopsy is the gold standard test for diagnosing CD, multiple serologic biomarkers are widely used (9). The anti-tissue transglutaminase (tTg) immunoglobulin (Ig)A test is included as an initial screening tool in the diagnostic algorithms of all recent guidelines (7, 10, 11) and has a reported specificity and sensitivity of >90% (12). The anti-endomysial IgA test is also included in some guidelines, and some studies have reported that it is the most specific test with up to 100% specificity and sensitivity (13). The American College of Gastroenterology (ACG) recommends diagnosing CD based on serologic testing for anti-tTg-IgA, with duodenal biopsy recommended as a confirmatory test (7). Recently, antibodies against synthetic deamidated gliadin peptide (DGP IgA and IgG) have been used to diagnose and monitor patients with CD (14).

A few studies have examined CD in adults and/or children in the United Arab Emirates (UAE), (15, 16) but evidence that can be used for a comprehensive assessment and characterization of these patients is lacking. Moreover, it is important to establish the sensitivity and specificity of the many available serologic tests.

This study describes the clinic-based prevalence of CD and the clinical, laboratory, and histopathologic characteristics of adults diagnosed with CD at the Cleveland Clinic Abu Dhabi in Abu Dhabi, the capital of UAE. The study also explored characteristics correlated with histopathologically confirmed CD and evaluated the performance of four serologic screening tests compared to the gold standard biopsy test for CD diagnosis.

## Materials and methods

### Data collection and patient grouping

We conducted a chart review of all patients screened for CD at the Digestive Diseases Institute (DDI) at Cleveland Clinic Abu Dhabi (CCAD). CCAD is a leading tertiary hospital in the UAE that has six centers of excellence including the DDI. The DDI have more than 15 gastroenterologists who are experts at their field and offer prevention, diagnosis and treatment of various digestive conditions.

Cleveland Clinic Abu Dhabi only accepts patients who are aged 14 years and above at the outpatient clinics. The chart review included patients who were suspected of having CD and referred for screening and diagnosis between January 1, 2015 to December 31, 2020. The CCAD follows the International Classification of Diseases (ICD) codes for documenting diseases in patients’ medical charts and follows the Current Procedural Terminology (CPT) codes for reporting the procedures and services provided to patients. Patients screened for CD were identified by ICD code K90.0 and the CPT codes 82784 and 83516. Patients diagnosed with CD from another healthcare facility and those who were on a gluten-free diet at the time of serologic testing were excluded.

Data extracted from medical charts included sociodemographic and anthropometric characteristics (age, sex, nationality, body mass index [BMI in kg/m^2^], and year of diagnosis), presenting symptoms (gastrointestinal manifestations or referral for other reasons), comorbidities, endoscopy, biopsy findings, hemoglobin [Hb], ferritin, vitamin B12, and vitamin D levels. Also, information on the serological testing results for the screened four CD-related serological biomarkers (anti-tTg IgA, anti-tTg IgG, anti-DGP IgA, and anti-DGP IgG) was also extracted.

According to the World Health Organization (WHO) guidelines (17), patients were categorized according to the body mass index (BMI) into underweight (<18.5 kg/m^2^), normal weight (18.5–24.9 kg/m^2^), overweight (25.0–29.9 kg/m^2^), and obese (≥30 kg/m^2^). Female patients with Hb > 12.0 g/dl and male patients with Hb > 13.0 g/dl were categorized as not anemic (18); the other categories were mildly anemic (Hb 11.0–11.9 g/dl for females and 11.0–12.9 g/dl for males), moderately anemic (Hb 8.0–10.9 g/dl for both females and males), and severely anemic (Hb < 8.0 g/dl for both females and males). Vitamin B12 concentration was categorized as above normal (>648 pg/ml), normal (128–648 pg/ml), or below normal (<128 pg/ml); vitamin D concentration was above normal (>150 ng/ml), normal (50–150 ng/ml), or below normal (<50 ng/ml); and ferritin level was above normal (>150 ng/ml), normal (15–150 ng/ml), or below normal (<15 ng/ml).

### Diagnosis of CD

At the DDI in CCAD, following physical examination and medical history, all patients presented with symptoms of CD or laboratory abnormalities suggesting malabsorption are considered as CD-suspected patients. Symptoms that could be related to CD include, but not limited to, abdominal pain, nausea, vomiting, diarrhea, constipation, change in bowel habits, bloating and gas, dizziness, brain fogginess, weakness and tiredness, skin rash, muscle and joint aches, weight loss, or growth retardation. All CD-suspected patients are offered a CD serological testing panel by their treating gastroenterologist. The testing panel is designed to screen for four CD-related serological biomarkers (tTG-IgA, tTG-IgG, DGP-IgA, DGP-IgG). In addition to the IgA level test to rule out its deficiency, patients who had IgA blood levels of less than 7 mg/dl were identified as having an IgA deficiency. At Cleveland Clinic Abu Dhabi, CD serologic testing is performed by enzyme linked immunosorbent assay (ELISA) kit supplied by QUANTA Lite h-tTG/DGP Screen by Inova Diagnostics. San Diego, USA. Blood samples were collected at CCAD laboratory and analyzed in the same laboratory by expert technicians. Antibodies tested by ELISA (anti tTG and anti DGP) are reported as positive at levels greater than 14.9 U/mL and antibodies levels of any value above 250 U/mL were reported as >250 U/mL. Patients who have anti-tTG IgA levels of above 14.9 U/mL were recognized as seropositive CD patients. To confirm a diagnosis of CD, all seropositive patients with a serologic titer ≥ 14.9 U/mL for at least one of these four sero-biomarkers were referred for an upper gastrointestinal endoscopy to evaluate histopathologic changes in their duodenal mucosa. Patients were instructed to continue on their normal gluten containing diet until the time of their scheduled endoscopy and biopsy. In each patient who underwent endoscopy and biopsy, four biopsies were collected from the duodenal mucosa with at least one from the duodenal bulb. The histopathologic examination was performed by an experienced pathologist and a Marsh score was assigned according to the severity of duodenal mucosal damage as follows; 0: normal duodenal biopsy, 1: mild damage that included intraepithelial lymphocytosis (25–100 enterocytes), 2: lymphocytosis with crypt hyperplasia, 3A: partial villous atrophy, 3B: subtotal villous atrophy, and 3C: total villous atrophy (19). Patients with CD were defined as those with a Marsh score > 1 (i.e., Marsh 2, 3A, 3B, or 3C), and those with a Marsh score of 0 or 1 were defined as having no histopathologic evidence of CD. Human Leukocyte Antigen (HLA) typing was used to exclude CD in patients with positive serology while equivocal small intestine histological finding. When typing is negative for both HLA DQ2 and HLA DQ8, CD was excluded.

### Statistical analysis

Categorical variables were summarized as frequencies and percentages and continuous data as means and standard deviations (SDs). The biomarker-based seroprevalence of CD was quantified. Also, the seroprevalence of CD based on seropositivity to at least one of the four sero-biomarkers was quantified. Histopathologically confirmed CD prevalence was determined based on the Marsh score for patients who underwent duodenal biopsy. We also quantified the proportion of patients with biopsy-confirmed CD who were positive for anti-DGP IgG and or anti-DGP IgA but not for anti-tTG IgA. The Chi-Square test was used to compare the categorical characteristics while *t*-test was used to compare continuous characteristics between patients who have biopsies confirming CD with patients whose biopsies didn’t confirm.

Compared to the golden standard biopsy test, we also evaluated the performance of the four serologic screening tests used to identify patients with CD. For each biomarker, the evaluated performance measures included sensitivity, specificity, positive predictive value (PPV), and negative predictive value (NPV).

Data were analyzed using SPSS Statistics v26.0 software (IBM, Armonk, NY, USA). *P*-values ≤ 0.05 (2-tailed) were considered statistically significant.

### Ethics approval

The study was approved by the research ethics committee of the Office of Clinical Research at Cleveland Hospital Abu Dhabi (REC #: A-2020-005), dated January 21, 2020.

## Results

**FIGURE 1:**
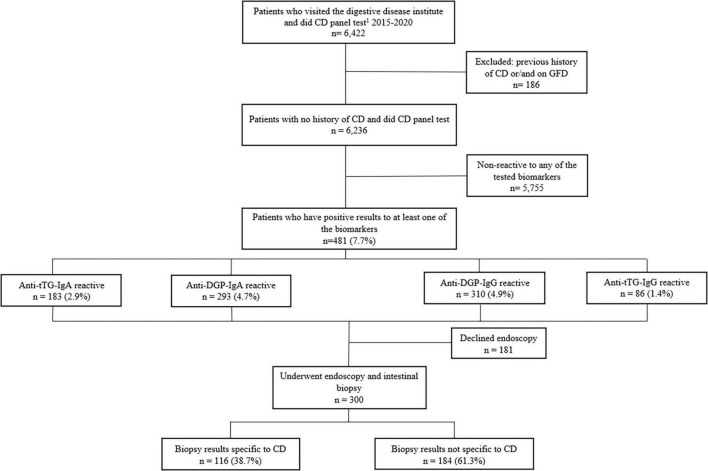
Flow chart of study population screened for the four CD-serologic biomarkers and underwent endoscopy examination. ^1^CD panel test includes tTG-IgA, tTG-IgG, DGP-IgA, DGP-IgG.

During the study period, 6,236 patients with no previous history of CD presented to the gastrointestinal diseases clinic with various symptoms and were screened for CD. Of the 6,236 patients, 481 (7.7%) patients were seropositive to at least one of the four tested sero-biomarkers. The sero-biomarker-based prevalence of CD was 1.4, 2.9, 4.7, and 4.9%, for Anti-tTG-IgG, Anti-TtG-IgA, Anti-DGP-IgA, and Anti-DGP-IgG, respectively (Figure 1). Of the 481 patients who were seropositive to at least one of the four CD biomarkers, only four had IgA deficiency, with levels < 7 mg/dl; only one of these patients had histopathologic changes indicative of CD in their duodenal biopsies.

The sociodemographic and clinical characteristics of the 183 (2.9%) patients who were serologically positive to Anti-tTG IgA, the most commonly used biomarker to screen patients for CD, are shown in Table 1. The mean age of patients who Anti-TtG-IgA seropositive was 32.1 ± 10.3 SD years, 72% of them were females, and 93.4% were Emirati. In those patients, overweight (28.7%) and obesity (24.7%) were common while 5.8% of patients were underweight. Anemia prevalence was 46.7%, 21.3% had Gastroesophageal reflux disease (GERD), 7.7% with autoimmune thyroid disease, and 5.5% (type 1) and 3.3% (type 2) were diabetic. Vitamin D deficiency was observed in 47.8% of the Anti-TtG IgA seropositive patients (Table 1).

**TABLE 1:** Socio-demographic characteristics, co-morbidities, and laboratory findings of the included 183 CD patients who are serologically confirmed using the Anti-tTG IgA biomarker.

	Serologic-positive patients to Anti-tTG IgA *n* = 183 (valid%)
**Sociodemographics**	
**Age (mean ± SD), years**	**32.1 ± 10.3**
14–29	82 (45.0)
30–45	81 (44)
>45	20 (11.0)
**Sex**	
Male	51 (28.0)
Female	132 (72.0)
**Nationality**	
Emirati	171 (93.4)
Non-emirati	12 (6.6)
**Marital status**	
Married	118 (68.0)
Single	55 (32.0)
Unknown	10
Body mass index, (mean ± SD kg/m^2^)	26.4 ± 6.04
Underweight (<18.5)	10 (5.8)
Normal (18.5–24.9)	71 (40.8)
Overweight (25.0–29.9)	50 (28.7)
Obese (≥30)	43 (24.7)
Missing	9
**Comorbidities**	
Gastroesophageal reflux disease (GERD)	39 (21.3)
Helicobacter pylori infection	29 (15.8)
Autoimmune thyroid disease	14 (7.7)
Diabetes type 1	10 (5.5)
Asthma	13 (7.1)
Diabetes type 2	6 (3.3)
**Laboratory findings**	
Hemoglobin, (mean ± SD g/l)	122.4 ± 19.5
Not anemic (*M* ≥ 130; *F* ≥ 120)	96 (53.3)
Anemic (males < 130; females < 120)	84 (46.7)
Mildly anemic (M, 110–129; F, 110–119)	34 (18.9)
Moderately anemic (80–109)	47 (26.1)
Severely anemic (<80)	3 (1.7)
Not tested	3
Vitamin B12 (mean ± SD pg/ml)	318 ± 121.4
Above normal (>648)	3 (3.1)
Normal (128–648)	93 (96.9)
Below normal (<128)	0
Not tested	87
Vitamin D (mean ± SD ng/ml)	54.5 ± 28.4
Above normal (>150)	2 (1.8)
Normal (50–150)	57 (50.4)
Below normal (<50)	54 (47.8)
Not tested	70
Ferritin (mean ± SD ng/ml)	44.6 ± 57.8
Above normal (>150)	11 (8.3)
Normal (15–150)	70 (53)
Below normal (<15)	51 (38.7)
Not tested	51
**Anti-tTG IgA**	
Reactive (titer ≥ 14.9 U/mL)	183 (100.0)
No-reactive (titer < 14.9 U/mL)	0
**Anti-tTG IgG**	
Reactive (titer ≥ 14.9 U/mL)	77 (42.1)
No-reactive (titer < 14.9 U/mL)	106 (57.9)
**Anti-DGP IgA**	
Reactive (titer ≥ 14.9 U/mL)	141 (77.0)
No-reactive (titer < 14.9 U/mL)	42 (23.0)
Anti-DGP IgG	
Reactive (titer ≥ 14.9 U/mL)	150 (82.0)
No-reactive (titer < 14.9 U/mL)	33 (18.0)
Stage of biopsy findings (performed for 134 patients)	
Marsh 0	17 (12.7)
Marsh 1	13 (9.7)
Marsh 2	1 (0.7)
Marsh 3A	29 (21.6)
Marsh 3B	58 (43.3)
Marsh 3C	16 (11.9)
**HLA genotyping (performed for 68 patients)**	
DQ2	49 (72.0)
DQ2/DQ8	13 (26.5)
DQ8	6 (12.2)

DGP, deamidated gliadin peptide; F, female; HLA, human leukocyte antigen; IgA, immunoglobulin A; IgG, immunoglobulin G; M, male; tTg, tissue transglutaminase.

Of the biopsy-tested three hundred patients 116 patients (38.6%) had histopathologic evidence of CD; these patients represent 1.9% of all patients who visited the gastroenterology clinic (Figure 1). Only one patient had a Marsh score of 2 and the others (99%) had a Marsh score ≥ 3. Twelve patients among the 116 patients (10.3%) who had biopsy-confirmed CD were found to be negative for Anti-tTG IgA while being positive for Anti-DGP IgG and/or Anti-DGP IgA. HLA typing was performed for seven of them and all had shown HLA DQ2 and/or DQ8 positive.

The sociodemographic and clinical characteristics of patients with biopsy-confirmed CD are shown in Table 2. The mean ± (SD) age of biopsy-confirmed patients was 33.8 ± (10.35) years. Most patients (84%) were ≤45 years old, females (72.4%), and of Emirati nationality (94%). The mean ± (SD) BMI was 26.6 ± (6.4) kg/m^2^, with nearly 6% being underweight and 53% being overweight or obese. Anemia was the most common associated comorbidity with a ratio of 52.6% (24.1% mildly anemic, 26.7% moderately anemic and 2 patients were reported with severe anemia). Among other comorbidities, (GERD) was reported in 21.6% and *Helicobacter pylori* infection in 16.4%. The mean ± (SD) vitamin B12 concentration was 319 ± (113.4) pg/ml and most patients (96%) had a concentration in the normal range. The mean ± (SD) vitamin D concentration was 52.1 ± (27.7) ng/ml; nearly half of patients (51.9%) had vitamin D deficiency. The mean (SD) ferritin level was 40.7 (53.3) ng/ml; 40.7% of patients had a ferritin level below normal (Table 2).

**TABLE 2:** Socio-demographic characteristics, co-morbidities, and laboratory findings of the serologically positive 300 patients and who were biopsy-examined.

	Confirming CD marsh > 1 *n* = 116 (valid%)	Not confirming CD marsh 0 and 1 *n* = 184 (valid%)	*P*-value^1^
**Sociodemographics**			
**Age (mean ± SD), years**	**33.8 ± 10.5**	**33.9 ± 12.7**	**0.949**
14–29	45 (38.8)	82 (44.6)	0.190^a^
30–45	53 (45.7)	65 (35.3)	
>45	18 (15.5)	37 (20.1)	
**Sex**			
Male	32 (27.6)	63 (34.2)	0.228^a^
Female	84 (72.4)	121 (65.8)	
**Nationality**			
Emirati	109 (94.0)	163 (88.6)	0.119^a^
Non-emirati	7 (6.0)	21 (11.4)	
**Marital status**			
Married	82 (70.7)	27 (69.2)	0.454^a^
Single	28 (24.1)	11 (28.2)	
Unknown	6 (5.2)	1 (2.6)	
Body mass index, (mean ± SD kg/m^2^)	26.6 ± 6.4	26.2 ± 6	0.759
Underweight (<18.5)	7 (6.2)	16 (8.7)	0.042^a^
Normal (18.5–24.9)	45 (40.2)	69 (37.5)	
Overweight (25.0–29.9)	30 (26.8)	63 (34.2)	
Obese (≥30)	30 (26.8)	36 (19.6)	
Missing	4	0	
**Comorbidities**			
Gastroesophageal reflux disease (GERD)	25 (21.6)	77 (41.8)	<0.001^a^
Helicobacter pylori infection	19 (16.4)	43 (23.4)	0.145^a^
Autoimmune thyroid disease	11 (9.5)	7 (3.8)	0.044^a^
Diabetes type 1	6 (5.1)	2 (1.1)	0.059^b^
Asthma	10 (8.6)	5 (2.7)	0.022^a^
Diabetes type 2	5 (4.3)	11(6.0)	0.524^a^
**Laboratory findings**			
Hemoglobin, (mean ± SD g/l)	120.7 ± 19.5	126 ± 19.2	0.021
Not anemic (*M* ≥ 130; *F* ≥ 120)	55 (47.4)	109 (59.6)	0.040^a^
Anemic (M: <130; F: <120)	61 (52.6)	74 (40.4)	
Mildly anemic (M: 110–129; F: 110–119)	28 (24.1)	43 (18.6)	0.184^a^
Moderately anemic (80–109)	31 (26.7)	39 (21.3)	
Severely anemic (<80)	2 (1.7)	1 (0.5)	
Not tested	0	1	
Vitamin B12 (mean ± SD pg/ml)	319 ± 130.9	327.7 ± 161.6	0.752
Above normal (>648)	3 (4.2)	3 (4.4)	0.345^a^
Normal (128–648)	68 (95.8)	63 (92.6)	
Below normal (<128)	0 (0.0)	2 (2.9)	
Not tested	45	91	
Vitamin D (mean ± SD ng/ml)	52.1 ± 27.7	67.5 ± 45.9	0.012
Above normal (>150)	0 (0.0)	4 (5.5)	0.023^a^
Normal (50–150)	39 (48.1)	43 (58.9)	
Below normal (<50)	42 (51.9)	26 (35.6)	
Not tested	35	111	
Ferritin (mean ± SD ng/ml)	40.7 ± 53.5	60.9 ± 111.2	0.119
Above normal (>150)	8 (8.8)	9 (9.6)	0.848^a^
Normal (15–150)	46 (50.5)	50 (53.8)	
Below normal (<15)	37 (40.7)	34 (36.6)	
Not tested	25	91	
Anti-tTG IgA			
Reactive (titer ≥ 14.9 U/mL)	104 (89.7)	30 (16.3)	<0.001^a^
No-reactive (titer < 14.9 U/mL)	12 (10.3)	154 (83.7)	
**Anti-tTG IgG**			
Reactive (titer ≥ 14.9 U/mL)	50 (43.1)	14 (7.6)	<0.001^a^
No-reactive (titer < 14.9 U/mL)	66 (56.9)	170 (92.4)	
**Anti-DGP IgA**			
Reactive (titer ≥ 14.9 U/mL)	96 (82.8)	93 (50.5)	<0.001^a^
No-reactive (titer < 14.9 U/mL)	20 (17.2)	91 (49.5)	
**Anti-DGP IgG**			
Reactive (titer ≥ 14.9 U/mL)	102 (87.9)	98 (53.3)	<0.001^a^
No-reactive (titer < 14.9 U/mL)	14 (12.1)	86 (46.7)	

DGP, deamidated gliadin peptide; F, female; IgA, immunoglobulin A; IgG, immunoglobulin G; M, male; tTg, tissue transglutaminase. ^1^*P*-value: assessing the difference between biopsy-confirmed and not confirmed patients with CD according to the measured characteristics (^a^obtained from Chi-square tests and ^b^obtained from the Fisher’s exact tests. For continuous variables the *p*-value was obtained from the two-sample *t*-test).

The proportion of patients with diabetes type 1, autoimmune thyroid disease, or asthma was statistically significantly higher in patients with biopsy-confirmed CD than in patients with negative biopsy. The proportion of patients with anemia or Vit D deficiency was also significantly higher among patients who had confirmed-CD (Table 2).

**FIGURE 2:**
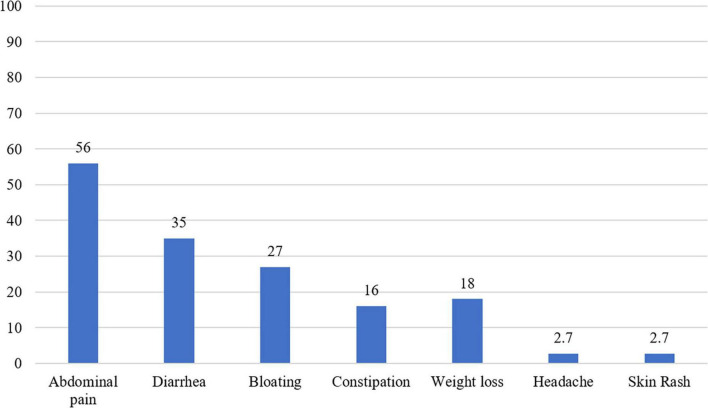
A histogram showing the distribution of symptoms among the seropositive celiac disease patients (*n* = 160).

Gastrointestinal complaints were the most common presenting symptom (∼80% of patients); abdominal pain was reported in 56% of patients, diarrhea in 35%, bloating in 27%, and constipation in 16%. Weight loss was the most common extraintestinal symptom and was reported in 34 patients (18%), while headache and skin rash were reported in five patients (2.7%) each (Figure 2).

Table 3 presents performance measures of the four serological biomarkers compared to the gold standard biopsy test. Anti-tTg IgA was the most sensitive biomarker (89.7%) for identifying true CD patients, with the highest NPV (92.8%); this was followed by anti-DGP IgG (87.9%) and anti-DGP IgA (82.8%), while anti-tTg IgG had the lowest sensitivity (43.1%). Our results also showed that Anti-tTG IgG has a high specificity of (92.4%), followed by Anti-tTG IgA with a specificity of (83.7%).

**TABLE 3:** Performance measures of the tested immunoglobulins compared to the biopsy finding (a gold standard test).

	Sensitivity	Specificity	PPV	NPV
Anti-tTG IgA	89.7%	83.7%	77.6%	92.8%
Anti-tTG IgG	43.1%	92.4%	78.1%	72%
Anti-DGP IgG	87.9%	46.7%	51%	86%
Anti-DGP IgA	82.8%	49.5%	50.8%	82%

tTG, tissue transglutaminase; DGP, deamidated gliadin peptide; IgA, immunoglobulin A; IgG, immunoglobulin G; NPV, negative predictive value; PPV, positive predictive value.

## Discussion

Our study population comprised a group of consecutive patients who were screened for CD at the Digestive Disease Institute (DDI) at CCAD. The Anti-tTG IgA serologically positive and biopsy-confirmed prevalence of CD in this population was 2.9 and 1.9%, respectively. Patients with biopsy-confirmed CD were more burdened with other comorbidities including diabetes type 1 and thyroid disorders. A significant proportion (10.3%) of the patients with biopsy-confirmed CD were seronegative to Anti-tTG IgA but seropositive to anti-DGP-IgA and/or Anti-DGP-IgG. These patients might have been missed if they were not screened for the three additional sero-biomarkers that are uncommonly used. The Anti-tTg IgA test had the highest sensitivity (89.7%) and NPV (92.8%) for identifying patients with CD.

The global population-based seroprevalence (based on positive results from tests for anti tTG and/or EMA antibodies) and biopsy-confirmed prevalence of CD have been reported as 1.4 and 0.7%, respectively (3). In the Kingdom of Saudi Arabia, a neighboring country to UAE, the reported seroprevalence in the general population was 1.5% (20). The slightly higher prevalence observed in our study population is attributable to the fact that our study is a clinic-based rather than a population-based study. The high biopsy-confirmed prevalence can also be explained by the fact that in our study patients who had reactivity to any of the four CD biomarkers were offered a duodenal biopsy, contrary to using anti tTG as a sole indicator for the disease.

The observed high prevalence (53.4%) of obesity and overweight among patients with CD is not surprising. This high prevalence reflects the high prevalence of obesity and overweight among general population in the UAE. In the Emirate of Abu Dhabi, about two-thirds (64.8%) of 12,346 adults aged ≥ 18 years were obese or overweight (21). However, the low prevalence of obesity and overweight among patients with CD compared to that in general population in the UAE (21) should be attributed to the fact that the study population in present study is patients who are presented with GIT-related symptoms. The most common gastrointestinal symptoms in our patients were abdominal pain, bloating, and diarrhea. Abdominal pain was also the most reported symptom among CD patients in Canada, Ireland, the United Kingdom, and Norway (22–24). Globally, the most common extraintestinal manifestation was anemia, with a prevalence ranging from 21 to 84% (25–28). The etiology of anemia in CD is multifactorial but mostly attributable to inadequate absorption of micronutrients in the proximal duodenum, which is typically damaged in patients with CD (29, 30). That malabsorption is more evident in patients who had atrophy in their duodenal mucosa as our results showed significantly lower levels of hemoglobin and Vit D among patients with villous atrophy. Nearly half (47.8%) of the patients with CD had Vit D deficiency. This observed high prevalence of Vit D deficiency stems from the documented high prevalence of Vit D deficiency among general Emirati population (72.0%) (21). In the present study, majority (93.4%) of patients were Emirati and nearly three-quarters (72.0%) were females. Several factors were reported to be associated with Vita D deficiency in the UAE and other Arab countries, including low exposure to sun light due to conservative dress style (21). It’s worth noting that diabetes type 1 and autoimmune thyroiditis were significantly higher among patients with confirmed CD and that supports previous reports which found a co-occurrence of multiple autoimmune diseases (4, 5).

A CD panel consisting of serologic biomarkers (anti-tTG IgA, anti-tTG IgG, IgA, Anti-DGP IgA, Anti-DGP IgG) was used to screen all of our patients. As in previous studies, we found that anti-tTg IgA had better diagnostic performance than anti-DGP IgA, anti-DGP IgG, and anti-tTg IgG (25, 31–33). The sensitivity of the four biomarkers tested in our study was comparable to what has been reported in the literature; while the specificity of Anti- DGP IgA/IgG was lower than the pooled specificity (96.9%) reported in a systematic review by Schyum and Rumessen (34).

The North American Society for Pediatric Gastroenterology, Hepatology, and Nutrition and ACG recommend DGP testing in addition to the anti-tTg IgA test to improve the accuracy of CD diagnosis in children < 2 years of age (7, 35). These tests were also the only diagnostic tests used in some studies of adult patients (32, 36–38). In our study, twelve biopsy-confirmed patients with CD were negative for anti-tTG IgA and positive for anti DGP-IgG and/or anti DGP-IgA. Given that only one of those patients had IgA deficiency, this result suggests that a more comprehensive serologic assessment of patients with suspected CD is warranted to improve diagnostic accuracy and avoid misdiagnosis and delays in treatment. It is worth noting that not all serologically positive patients in our study underwent endoscopy, which made it difficult to establish a confirmed diagnosis and reduced our sample size, which could explain the low specificity of the anti-tTG IgA test in our cohort.

One of the limitations in our study is that although strict criteria were followed for biopsy confirmation of CD at the DDI which include increased intraepithelial lymphocytes, villous atrophy and crypt hyperplasia, there are some other intestinal disorders share similar histopathological features with CD. Parasitic infections, H-pylori gastritis and other immune inflammatory conditions might lead to histopathological changes similar to that of the CD (39). However, this potential limitation should not impose any changes on the presented findings as none of the patients had any medical information related to parasitic infections and only three patients diagnosed with H. pylori. This assumes that the observed histopathological change is highly likely to be CD-related, particularly with the documented seropositivity to CD. Another potential limitation is that our study was limited by the information available in medical charts that was not originally collected for research purposes. As this study utilized already-collected data in medical records, there was no screening performed for patients with a high risk of developing CD, patients who have a previous diagnosis of CD were excluded, and two-thirds of the serologically positive patients declined to undergo endoscopy for histopathological investigation. Additionally, reviewing medical charts from a single center in one of the seven emirates in the UAE imposes a major limitation on the generalizability of the present findings to general populations in the UAE. Finally, 26 patients who had Anti-TtG IgA levels of above 250 U/mL have denied endoscopy and biopsy which might potentially results in an underestimation of the disease confirmed cases. Despite these limitations, there are multiple worth mentioning strengths to this study. First, the study adds to the data on CD in the UAE by including adult patients from a major and referral digestive disease clinic. Specifically, we carried out a comprehensive analysis of medical chart data to obtain a broad view of the clinical spectrum of CD in the UAE. Second, all adult patients with suspected CD based on clinical presentation were screened using the four serologic biomarkers and confirmed based on histopathological investigation following established guidelines. Finally, this is the first study in the UAE to explore the performance and accuracy of the serological testing panel that screens for four serological CD-related biomarkers and to provide evidence-based recommendations.

## Conclusion

Three and two of every 100 patients were serologically (anti-tTG-IgA) and histopathologically diagnosed with CD, respectively. Although anti-tTg IgA is included in most CD diagnostic algorithms as an initial screening tool, our results showed that 10.3% of biopsy-confirmed patients would have gone undiagnosed by relying solely on this test. Thus, comprehensive serologic screening for all four serologic biomarkers is necessary so patients with CD won’t be missed if only the traditional TtG-IgA screen is followed.

## Data availability statement

The raw data supporting the conclusions of this article will be made available by the authors upon providing proper justification and eliciting necessary approvals from the ethics committee.

## Ethics statement

The studies involving human participants were reviewed and approved by the Office of Clinical Research at Cleveland Hospital Abu Dhabi (REC #: A-2020-005). Written informed consent for participation was not required for this study in accordance with the national legislation and the institutional requirements.

## Author contributions

AS and RHA conceived and conceptualized the study. AHA and AA extracted the data. AHA performed the data analyses and drafted the first version of the manuscript. All authors contributed to the data extraction strategy, data analysis plan, findings interpretation, reviewed, and approved the manuscript.
